# Supplementary material for: Are carbon emissions trading and green financial instruments synergistic? -Comprehensive quantitative research based on content analysis
Source: PLoS One. 2024 Mar 7;19(3):e0298601. doi: 10.1371/journal.pone.0298601 (PMC10919601; doi:10.1371/journal.pone.0298601)
Supplement: S1 Data — (ZIP) [file pone.0298601.s001.zip › Data/地级市Z_F工作报告.zip!/┴╔─■/░░╔╜╩╨/└┤╫╘╣1⁄2╓┌║┼í╛┬φ┐╦╩2╛▌═°í┐_2008.docx]

各位代表：

现在，我代表市第十三届人民政府向大会报告工作，请予审议，并请市政协各位委员和其他列席人员提出意见。

一、过去五年主要成就及2007年政府工作回顾

市第十三届人民政府在市委的正确领导下，在市人大依法监督和市政协民主监督下，深入贯彻落实科学发展观，突出加速经济隆起、构建和谐鞍山主题，围绕实现“总量倍增、位次前移、追赶沈大、殷实和谐”的奋斗目标，全面推进经济与社会、城市与农村、人与自然的统筹协调发展，“十五”计划圆满完成，“十一五”规划顺利实施，振兴鞍山老工业基地取得重大进展。

――全市经济实力快速提升。五年中，地区生产总值增长1倍以上，年均增长15.4%；地方财政收入增长2.1倍，年均增长25.2%，其中一般预算收入增长1.5倍，年均增长20.5%；累计完成全社会固定资产投资1561亿元，年均增长26.2%。鞍山综合实力在全国百强城市排名由2002年第50位，2004年升至第44位，2006年跃升至第34位。

――改革开放全面深化。国有企业改制任务全面完成，鞍钢钢铁主业实现整体上市，地方国有企业和集体企业历史遗留问题基本解决。对外开放全面拓展，累计利用外商直接投资11亿美元，比前五年增加3倍；引进内资840亿元，比前五年增加4.8倍。

――经济结构不断优化。“一个基地、三大产业”主导地位更加突出，现代农业和服务业加速发展。国有经济不断做大做强，鞍钢500万吨精品板材基地全面建成。民营经济发展迅速，占全市比重由2002年的48%提高到51.4%。

――城乡环境明显改善。城市主要道路全面改造，农村实现村村通油路。全市森林覆盖率由2002年45.3%提高到46.4%，城区绿化覆盖率由34.7%提高到36.9%。先后成为国家卫生城市、全国畅通工程A类管理一等模范城市。

――社会建设全面加强。城乡义务教育全面实现，农村实现“两免一补”。城乡医疗卫生服务体系更加健全，社会保障体系更加完善，就业再就业矛盾有所缓解。成为全国科技进步先进市和全国民族团结模范市，蝉联全国双拥模范城市四连冠。

――人民生活水平明显提高。城市居民人均可支配收入五年增长90%，年均增长13.7%；农民人均纯收入五年增长62%，年均增长10.1%。城市人均住房建筑面积由19.1平方米增加到21.5平方米；农村人均住房面积由22.5平方米增加到26.6平方米。

各位代表，过去的五年，是全市上下开拓创新，鞍山实现历史性跨越的五年；是城乡面貌发生巨大变化，人民群众得到实惠较多的五年；是贯彻落实科学发展观，为未来可持续又好又快发展奠定坚实基础的五年。五年发展取得的辉煌成就，激发了全市人民加速经济隆起、构建和谐鞍山的昂扬斗志，让全市人民看到了鞍山老工业基地全面振兴的美好未来！

2007年，我们按照市第十次党代会的总体部署和市委十届二次全会提出的“扭住一个核心，突出两大主题，实施三个同步推进”的总体要求，坚持一手抓发展、一手抓民生，一手抓城市、一手抓农村，一手抓空间、一手抓项目，全面超额完成市十三届人大五次会议确定的各项任务，鞍山正在步入又好又快发展的新时期。预计全年实现地区生产总值1350亿元，增长16.3%；地方财政收入首次突破100亿元，达到103.2亿元，增长45.2%，其中一般预算收入完成74.5亿元，增长32.6%；全社会固定资产投资477亿元，增长26%；实际利用外商直接投资2.78亿美元，增长1.05倍；引进内资350亿元，增长30.5%；全年银行业机构各项贷款余额增加123亿元，达到646亿元，增长23.5%，创历史新高；规模以上工业实现增加值515亿元，增长21%，实现利润157亿元，增长20.3%；社会消费品零售总额实现298.5亿元，增长17.5%；外贸出口23.6亿美元，增长11%。城市居民人均可支配收入达到12856元，增长19.5%；农民人均纯收入达到6125元，增长16%，均为十年来收入增长最快的一年。

（一）积极抢抓机遇，破解制约鞍山发展的空间瓶颈

进一步完善城市规划和产业布局规划，依法全面拓展发展空间，为鞍山大发展、快发展抢占先机。

“一带五区”工业发展空间形成。积极抢抓机遇，通过农村征地和村屯移迁，将达道湾污染地变为建设用地，依法拓展了工业空间。在城市西部，以腾达大道为纽带，由北向南建设灵山、达道湾、高新区（西区）、宁远、汤岗子五个工业规划区。由政府投资建设道路、污水处理和供气、供暖、给排水管网等基础设施，引进国电、东电投资建设供电和供热设施，五个工业规划区内的基础设施基本建成。达道湾规划区建成鞍刘路、鞍旗路、钢西路等16条主要干道，形成“四纵六横”路网，一个现代工业集聚地正在形成；灵山规划区建成东建街、曙光路、灵山路等7条道路，形成“三纵两横”路网，初步实现园区化、城市化；高新区新拓展空间4.5平方公里，主要经济指标连续三年保持35%以上增长，在全国排名由第52位跃升至第33位；汤岗子规划区基础设施实现“七通一平”。截止2007年底，工业规划区内已有442个项目开工建设，121个项目竣工投产。“一带五区”正在成为鞍山重要的经济增长点。

“一区四带”商业发展空间形成。通过市区企业搬迁、居民动迁和城乡结合部村屯移迁，有效拓展商业发展空间。在千山西路和人民路之间规划建设四达批发市场集中区；在胜利路、解放路建设精品商业带；在鞍刘路规划建设东北钢铁物流产业带；在千山西路、解放西路建设汽车贸易产业带；在千山东路规划建设文化旅游产业带。在市区按商业布局规划建设一批大中型商业设施。全市形成了块状化、带状化、网格化、点状化的商业发展新格局。截止2007年底，市区内已有158个大型商业项目开工建设和竣工营业。

“集中连片”民居发展空间形成。结合棚户区和危房改造，集中建设回迁房，连片开发商品房。解放路富兴小区、胜利路健身新区、立山双兴绿园等高档次回迁住宅拔地而起；睿达花园新城、阪芙小镇等农民新居基本建成；世纪峰景、金色家园、国际华府、凯圣凡尔赛等高标准商业住宅全面建设。全市建成风格各异、布局合理、功能齐全的现代住宅小区142个。

（二）大力推进结构调整，积极转变经济增长方式

坚持以项目为核心，以项目攻坚年为牵动，促进结构优化升级。全市“四个一批”项目总数达到2000个，总投资2700亿元。新开工建设项目800个，竣工投产300个。新增规模以上工业企业400家，总数突破1400家;新增规模以上商业企业60家，总数达到524家。

积极开展招商引资，项目攻坚年取得丰硕成果。全面开展了以攻坚大项目、产业集群项目和现代服务业项目为主要内容的项目攻坚年活动。积极争取中直、本地民营、外埠民营和境外企业资本投资鞍山。引进百亿元项目取得重大突破。全年引进和开发销售收入超百亿元项目11个。鞍钢有取向硅钢、中冶东建百万吨管材、宝得钢铁260万吨精品型材、中航科技大型中央空调和特种车、中船重工船舶用钢加工配送基地、百万吨紧固件产业园、石化产业集群、海城纺织工业园腾龙一期1.5万吨帘子布等8个项目开工建设；国电集团投资100亿元建设8×300MW发电机组热电联产和鞍钢煤焦油深加工等2个项目完成选址；投资3亿美元的台湾烨联30万吨冷轧不锈钢项目即将签约。鞍山特色产业集群加速发展。洽谈推进集群项目806个，总投资1225亿元，鞍山正在形成冶金及矿山设备、钢结构、船舶零部件、铸锻件、紧固件等产业集群30个。现代服务业迅猛发展。四达批发市场集中区已有东北化工建材城、瑞盛国际汽配城等31个项目即将进驻或开工建设；鞍刘路东北钢铁物流产业带已有中船重工船舶用钢配送加工中心、鞍钢板材剪切配送中心等项目开工建设；胜利路精品商业带引进新世界百货、大商新玛特、兴隆大家庭、家乐福、乐购、香格里拉大酒店等30余家国内外知名企业落户；千山西路汽车贸易产业带新增奔驰、宝马、福特、现代等4Ｓ店11家；全面盘活了国贸大厦、南山宾馆等多处商业闲置资产。全年接待境内外旅游者1200万人次，增长26.6%；旅游综合收入86亿元，增长27.7%。

坚持“双轮驱动”，以鞍钢为代表的国有经济和以民营企业为代表的地方经济竞相发展。全面深化“地企融合、共兴共荣”理念，积极解决鞍钢、中冶东建等中省直企业上项目用地问题，全面改善鞍钢、中冶东建等周边环境。继500万吨精品板材基地投产达产后，鞍钢新一届领导班子进一步加大在鞍投资力度，相继实施了有取向硅钢、煤焦油深加工、1450冷轧、100米重轨、60万吨线材、无缝管改造及板材剪切配送等一批重大项目。鞍钢全年销售收入突破800亿元，增长15%；实现工业增加值330亿元，增长19.5%；实现利润125亿元，增长13.2%；实缴税金96.2亿元，增长39.5%；完成固定资产投资139亿元，增长25.4%。鞍钢为鞍山发展做出巨大贡献。中冶东建产业园全面形成，正在打造百亿元企业。中钢热能、中冶焦耐、中冶北方等中直企业全面发展。不断优化民营经济和中小企业发展环境。荣信股份成功上市，结束了辽宁四年、鞍山十年没有企业上市的历史；森远、聚龙等5家企业成为国家行业标准制定单位；“鞍轮”成为全国驰名商标；后英、西洋、宝得、衡业、华冶等众多民营企业迅速壮大。全年民营经济实现增加值694亿元，增长18.2%；实缴税金48.8亿元，增长18%。

高度重视节能减排，可持续发展能力全面增强。大力开展结构、技术、管理节能减排。积极引进和发展低能耗、少污染、深加工、产业链条长、高产出、高科技项目。新发展装备制造项目234个，装备制造业占规模以上工业比重达到9%，比上年提高3个百分点；新发展钢铁深加工项目56个，钢材本地加工能力由不足100万吨增加到200万吨；新发展矿产品深加工项目92个，矿产品深加工率由上年35%提高到40%。高新技术企业发展到194家，实现高新技术产品产值300亿元，增长30%。鞍钢建成国内首台300MW燃气蒸汽联合循环发电机组（CCPP），实现了高炉煤气零排放。全市实施节能减排改造工程84项，依法重组、改造、关停“五小”企业255户，万元GDP综合能耗下降5.3%。

（三）加大政府投入，新农村建设迈出重大步伐

坚持一手抓城市、一手抓农村，在领导精力、资金投入、项目安排和改善民生等方面全方位向农村倾斜。全年财政投入“三农”资金超过6亿元，增长52%。

设施农业发展实现新突破。把发展设施农业作为农民增收的有效途径。在鞍羊路沿线实施政府扶持政策，新建标准化温室大棚每栋补贴2000元，新建标准化养殖棚舍每栋补贴2万元。贯穿千山区、海城市、台安县7个镇、45个村的鞍羊路设施农业示范带基本形成，沿线建设了宁远镇张忠堡蔬菜花卉日光温室小区、高坨镇三合肉鸡养殖小区、黄沙镇万头奶牛养殖基地、金桥蛋鸡养殖场、温州现代农业园、新开河镇棚菜种苗繁育基地等31个设施农业重点项目，发展温室大棚和标准化养殖棚舍1.3万栋。全市新增设施农业10万亩，总数达到50万亩。设施农业镇发展到23个，设施农业村发展到120个。与设施农业相配套，由政府出资，新建了海城中小镇蔬菜批发市场；市政府出资，为乡镇聘请农业技术人员266名、兽医979名；建成45个乡镇动物卫生监督所和现代农民远程科技培训视频网络系统。通过发展设施农业，全市农民人均增收370元。

县域经济发展实现新突破。市政府加大投入，帮助各县（市）区解决县域经济发展难题。出资7000万元，支持海城新建4个变电所，解决了因电力不足严重制约发展的瓶颈；出资2000万元，支持台安发展设施农业，大力推进农产品深加工；出资2000万元，支持岫岩矿产资源勘探，变资源优势为产业优势；支持千山区加快城市化和城镇化，全区74个村正在改造24个。支持三县（市）各1000万元，用于工业规划区基础设施建设，县域工业规划区发展到16个，新上工业项目296个。全年县域经济实现增加值574.2亿元，增长18.4%；县域财政一般预算收入18.6亿元，增长26.8%。

解决农村民生问题实现新突破。切实解决农民饮用水安全问题，政府投资9403.8万元，全市又有30.7万农民用上安全自来水。创新解决被征地农民保障问题，建立了失地有新房、失地有保障、失地有工作、失地有补偿“四位一体”的保障机制。在全国第一个出台了由政府出资，被征地农民女50岁、男55岁享受与城市低保标准挂钩的基本养老保障制度。新建和维修3301户农村困难群众住房，改造农村敬老院23所。农村低保标准由每人每年600元提高到1000元。政府出资新建10所农村寄宿制学校，为海城、台安、岫岩各配备10台中小学校车，为全市433所农村中小学建成现代远程教育工程。完善农村医疗卫生服务体系，出资2000万元，改造29所乡镇卫生院，为70所乡镇卫生院购置设备502台，为岫岩10个偏远村建设了标准化卫生室，在省内率先实现乡镇卫生院全部达到国家规定标准。投资5722万元，修建农村公路159公里。更新鞍山至海城、台安长途客车100台。出资2369万元，实施“千村绿化”工程，绿化行政村796个。新建和维修了100个村委会办公用房。为200个行政村配备了健身器材。农村新能源普及率达到28%。

（四）有效破解“钱从哪来，人往哪去”难题，深化企业改革取得重大进展

我们满怀对广大工人群众的深厚感情，着力解决地方国有和集体企业历史欠账问题和以鞍钢附企为重点的中省直厂办大集体改革发展问题。通过系统性安排，协调性推进，整体性突破，根本性解决，有效破解了“钱从哪来，人往哪去”两大改革难题。

地方国有企业和市属厂办集体企业历史遗留问题基本解决。推进企业东搬西建，通过土地置换方式，利用级差地价获得收益，解决了140户企业改革发展的资金问题。采取政府收购方式盘活企业资产，92户破产企业改革成本全部支付。政府直接出资全额支付国有“壳”企业职工“两险一金”，支付集体“壳”企业职工全额“两险”和50%补偿金。政府出资1亿元为138户困难企业4.5万名退休职工补贴了取暖费。在四个城区设立离退休职工托管中心，4.6万名企业退休职工全部与原企业脱钩，实行统一的社会化管理。到2007年底，地方741户国有企业和市属245户厂办集体企业剩余的20亿元改革成本全部支付。

推进鞍钢附企改革取得新进展。按照“发展、就业、改革、保障”八字方针，与鞍钢齐心协力，积极稳妥地推进鞍钢附企改革发展。支持鞍钢附企做大做强。政府出资2亿元，设立鞍钢附企发展资金。建设灵山和达道湾附企工业园，实现附企搬迁整合、改造升级。全年实现销售收入23.5亿元，增长27%。推进附企职工转企就业。设立附企再就业平台，为附企职工免费提供就业培训和就业服务，有7000多名附企职工重新就业和转企就业。规范推进附企改革。首批选择12户企业进行改革试点，与原企业解除劳动关系的转企就业职工，各种保险全部接续，政府垫资先期支付50%经济补偿金。切实保障职工合法权益。将鞍钢附企退休职工全部纳入城市合作医疗。政府借资2000万元，全面解决了附企职工丧葬费、遗属费等历史遗留问题。对困难家庭和重病职工实行救助，全年发放救助金9350万元，救助困难职工1.5万人。鞍山做法得到国家国资委和省委、省政府的充分肯定。

（五）全面加强城市建设与管理，城市面貌发生较大变化

编制完善了鞍山市西部和南部路网及胜利路全线景观等30个专项规划。科学推进了城市改造，为创建全国文明城市奠定了良好基础。

城市基础设施进一步完善。投资8亿元，新建鞍山500千伏、达道湾66千伏等4个变电所。出资2.7亿元，改造供暖、供水、供气等地下管网105公里。出资6亿元，新建和改造千山西路、千山中路、矿工路和四达大道等城市主次干道28条。深营路西线隧道建成通车，形成鞍山一环线，结束了鞍山没有环路的历史。完成沈大高速公路鞍山站出入口改造，千山西路、解放西路、灵山路、鞍辽路全部改成双向8车道，不仅改善了城市交通，同时也为达道湾、灵山等工业规划区发展提供了重要支撑。第二污水处理厂二期开工建设。全面改造了二一九、孟泰、永乐、烈士山和人民公园，新建双潭公园和沙河文化公园。改造了30个居民小区和15条街巷路。每个社区至少建有一处健身广场。新增城市绿地70万平方米。

城市社会功能不断健全。把完善社会功能纳入城市规划和改造之中，作为土地出让和开发的必备条件。规划建设了停车场72处、派出所26个、幼儿园12个、社区“一站四室”41个、社区医疗服务中心10个、公厕70个、农贸市场50个。

城市环境全面整治。大力开展以“整治经营秩序、规范城市管理”为主要内容的综合整治行动，全面规范了餐饮、土木销售、铝塑和铁艺加工等行业经营行为，取缔违章经营和无证业户3000多家，经营秩序逐步规范，城市环境有所改善。

（六）强化以民生为重点的社会建设，改革发展成果全民共享

为全市人民办的18件实事基本完成，一大批人民群众最关心、最直接、最现实的问题得到有效解决。

实现群众安居有新突破。采取政府主导、市场化运作方式，大力度实施了棚户区和危房改造，又快又好地建设了回迁房。实施最优惠的政策，原地安置动迁户，回迁房面积全国最大，回迁超面积收费全省最低，3404户低保家庭免费入住新房，2117户低保边缘户通过民政救助保证回迁。城区累计拆迁棚户区和危房190万平方米，开工建设回迁房255万平方米，动迁居民63%已经回迁。5.1万户家庭住房改善，受益群众14.5万人。鞍山城区已经彻底结束了棚户区和危房区历史，闯出一条百姓、企业、政府三方共赢的棚户区和危房改造新路子。

促进就业再就业有新成绩。大力开辟就业源，全年实名制就业13.5万人。动态消除“零就业家庭”1.5万户,50%以上的“零就业家庭”实现双就业，其中棚户区“零就业家庭”全部实现双就业。开工建设了市劳动力市场大厦。设立大学生创业资金500万元、就业援助资金300万元，帮助大学生就业，毕业生就业率达到75%以上。全市城镇登记失业率为4.3％。

完善社会保障体系有新进展。依法推进社会保险应保尽保和应缴尽缴。全年新增养老保险3.8万人、城镇职工基本医疗保险7.5万人、工伤保险4.1万人，征收社会保险费21.5亿元，增长32.7%。农村新型合作医疗覆盖面达到95%以上。城市合作医疗参保人数达到5.8万人。城市低保对象由上年16.9万人减少到13.4万人。慈善事业快速发展。多层次的社会救助体系不断完善，走出了一条由临时救助走向制度救助的新路子。

改善群众日常生活条件有新成效。政府出资近4000万元，通过拍卖取得北方供暖公司产权，并又追加投资5000万元对其管网进行全面改造，解决了100万平方米供暖质量差问题。鞍钢出资3亿元，对其供暖锅炉和管网进行了改造。北美公司完成了铁西区100万平方米供暖分户和二热电2台供暖锅炉改造，改善了200万平方米用户的供暖条件。改造供水管网和设施，扩大供水范围，部分城区供水质量得到改善。改造燃气进户管线5513处。积极争取鞍钢、辽河油田的支持，解决供气需求问题。大力发展公共交通，政府出资6000万元，新增公交运营车辆200台，公交线路不断延伸和完善。

解决疑难信访案件有新成果。政府每周至少召开两次信访专题会议，积极解决信访实际问题，全年解决企业拖欠工资、社保、供暖、弃管房等重点疑难信访案件400余件，上访数量明显减少，社会保持基本稳定。

加强社会管理有新提高。建立健全市、县（区）、街道、社区四级应急管理体系。区划地名首次实现“六号合一”，农村结束无门牌历史。全面加强了食品药品市场监管。开展猪肉质量安全专项整治行动，城市定点屠宰率100%，乡镇超过95%。加强安全生产工作，整改各类消防和安全隐患5016处。创建全国综合治理先进市，全面强化公安系统“三基”建设，严厉打击了各类犯罪活动，平安钢都建设迈出新步伐。

发展社会各项事业有新进步。改造薄弱学校20所。为100所中小学统一配备了体育器材，建设了实验室和图书馆。全年校际间师资交流超过100人。新建了市博物馆和群众艺术馆，改造了市图书馆、青少年活动中心。完善城市公共卫生服务体系，社区医疗覆盖面达到100%。实行了70周岁以上老人免费乘坐公交车。建设了市中心医院老干部病房。人口与计划生育、新闻出版、广播电视、妇女儿童、民族、宗教、外事、侨务、对台、接待、史志、档案、气象、地震、人防、民兵预备役等工作都有新成绩。

（七）推进依法行政，政府自身建设不断加强

自觉接受监督。认真贯彻《监督法》。自觉接受市人大及其常委会的依法监督、市政协民主监督、人民群众的社会监督和新闻媒体的舆论监督。主动向人大及其常委会报告工作，与人民政协民主协商。111件人大代表建议和492件政协提案办结率、见面率和满意率均为100%。市政府首次被省人大、省政府授予代表建议办理工作先进单位。

严格依法行政。严格按照法定权限和程序履行政府职责。城市改造和规划区建设严格执行城市总体规划。在农村土地征用、城市居民动迁、城市建设与管理、社保资金管理等方面，严格按法定程序和规定执行。综合行政执法改革走在全省前列。市政府被省授予依法行政工作先进单位。

强化制度约束。对经营性土地和工业用地全部实现净地挂牌出让，对政府采购、项目招投标及国有资产出租转让等经济活动全部按程序规范操作，对行政收费和市场服务收费实行定值管理，从制度上保证政府行为的公开、公平、公正。

做到自律用权。政府班子及成员自觉遵守廉洁自律各项规定，坚决执行中纪委的“八个禁止”，始终坚持政府班子集体议事决策制度。积极推行政务公开，鞍山被国务院确定为全国政务公开示范市。

各位代表，经过全市上下的不懈努力，我们有效破解了制约鞍山发展的一系列重大问题，鞍山已经进入到本地、域外、境外资本共同投资发展的新时期，进入到城市与农村、经济与社会又好又快发展的新阶段。这些成绩的取得，得益于市委的正确领导和市人大、市政协的大力支持，得益于全市各族人民的共同奋斗，得益于鞍钢等中省直单位和民营企业的巨大贡献，得益于各地区、各部门的辛勤工作，得益于全市上下只争朝夕、埋头苦干。在此，我代表市政府向各位代表和委员，向全市各条战线的广大干部群众，向各民主党派、工商联、人民团体、中省直单位、驻鞍部队、武警官兵和关心支持鞍山发展的老领导、老同志，向所有为鞍山发展做出贡献的海内外朋友，表示衷心的感谢和崇高的敬意！

各位代表，在总结成绩的同时，我们也清醒地看到鞍山经济社会发展中还存在许多问题。主要是战略性、牵动性的大项目较少，开放型地区经济总体水平不高；产业结构调整还不到位，钢铁和矿产品深加工产业链条短，传统服务业比重仍然较大，生产型服务业发展相对滞后，现代农业比重低；经济增长的资源环境代价过大，转变经济发展方式的任务相当艰巨；县域经济发展不快，城乡发展不平衡问题仍很突出；一些关系群众切身利益的问题还没有得到根本解决，一些影响社会稳定和谐的因素仍然存在；安全生产和消防安全亟待加强；发展环境还需不断改善，政府工作效率有待进一步提速，政府自身建设亟待改进。这些问题需要我们今后采取有力措施，认真加以解决。

二、今后五年奋斗目标及2008年政府主要任务

今后五年是全面振兴鞍山老工业基地的重要时期。我们将紧紧抓住东北振兴和沿海开放“双重机遇”，不断开创鞍山老工业基地全面振兴的新局面。今后五年政府工作指导思想是：全面贯彻党的十七大精神，以邓小平理论和“三个代表”重要思想为指导，深入贯彻落实科学发展观，突出加速经济隆起、构建和谐鞍山主题，围绕实现“总量倍增、位次前移、追赶沈大、殷实和谐”奋斗目标，深化改革，扩大开放，优化产业结构，转变经济发展方式，全面推进经济建设、文化建设、社会建设和城乡一体化，实现全市经济社会好中求快发展，全面完成“十一五”规划，在省内率先实现全面振兴。

今后五年的奋斗目标：全市地区生产总值年均增长15%。地方财政一般预算收入年均增长15%。固定资产投资年均增长20%。实际利用外商直接投资和引进内资年均分别增长20%以上。建成全国重要精品钢材基地，鞍钢进入世界500强。科技对经济增长贡献率超过60%。服务业占经济总量比重超过45%。设施农业占农业比重超过30%。城镇登记失业率控制在4.5%以内。城市居民人均可支配收入和农民人均纯收入年均分别增长10%以上。社会保障体系、社会管理体系、公共卫生服务体系和公共行政服务体系更加完善。万元GDP能耗年均递减4.5%以上，城乡生态环境明显改善。鞍山在全国百强城市中位次前移，成为国家环保模范城市、全国文明城市和国际健康城市。

2008年，我们将认真贯彻市委十届四次全会精神，“围绕一个核心、实现两个融入、推进三大任务”，即以项目升级为核心，积极融入辽宁“五点一线”沿海经济带和中部城市群经济区，突出抓好推进城乡一体化、创建全国文明城市、改善民生三大任务，继续坚持一手抓发展、一手抓民生，一手抓城市、一手抓农村，一手抓空间、一手抓项目，全力推进经济社会发展好中求快，紧中求进。全年主要预期目标是：实现地区生产总值增长15%；地方财政收入增长20%，其中一般预算收入增长15%；实际利用外商直接投资增长20%，引进内资增长20%；全社会固定资产投资增长20%；地方外贸出口增长10%以上；社会消费品零售总额增长14.5%；城市居民人均可支配收入和农民人均纯收入分别增长10%以上；万元GDP能耗下降5.3%，主要污染物排放量平均减少5%。

（一）大力开展项目升级年活动，全面优化经济结构

全面开展项目升级年活动。积极融入辽宁“五点一线”沿海经济带和中部城市群经济区。推进腾达大道南下，逐步实现沿线各工业规划区的有机连接，形成鞍山工业主体功能区。加速城市和县域工业向工业规划区集中，服务业项目向城区、城镇集中。继续坚持一手抓空间、一手抓项目，大力推进项目规模、质量、效益、结构和环境全面升级，全年力争开工建设项目800个，竣工投产300个，新增规模以上企业400家。

推进项目规模升级。注重项目单体向大升级，整体向集群升级。继续谋划和引进重大项目，力争再引进、开发和培育10个销售收入超百亿元项目，10个50亿元项目，50个10亿元项目，200个亿元项目，规模以上项目超过2000个。力争促成1——2个在全省乃至全国有较大影响，对全市经济社会具有重大牵动作用的特大项目。围绕30个产业集群，加大招商引资力度，继续引进集群项目，全面做大做强钢结构、船舶零部件、铸锻件、紧固件、金属家具厨具和石化、煤化工深加工等产业集群。

推进项目质量升级。重点引进和发展低能耗、低污染、高附加值、科技含量高、符合国家产业政策的好项目，吸引拥有先进技术、处于行业领先地位的龙头企业落户鞍山。全年新增省级高新技术企业力争达到10家，总数突破200家。高新技术产品产值达到370亿元，增长25%以上。

推进项目效益升级。坚持速度、质量、效益相统一原则，注重提高项目的经济效益和社会效益。着重引进和开发经济效益好、财力贡献大的项目；引进和开发能够强市壮县富民、能够充分吸纳就业的项目；引进和开发提高产品附加值、提升市场竞争力的项目，实现以较少的投入获取更大的效益。

推进项目结构升级。政府投资4000万元，设立“结构调整专项资金”，专门用于优化产业结构。大力引进和开发设施农业和农业产业化项目。大力引进和开发现代服务业项目。以建设千山路文化旅游产业带为重点，大力引进和开发文化旅游项目，做大做强文化旅游产业。大力引进和开发先进装备制造项目，装备制造业占工业比重力争由9%提高到12%；注重引进和开发钢铁深加工项目，钢材本地加工能力力争由200万吨提高到300万吨；注重引进和开发矿产品深加工项目，矿产品深加工率力争由40%提高到47%。

推进项目环境升级。牢固树立“政府竞争环境、企业竞争市场”理念。坚持亲商、护商、助商，大力整改机关不作为、乱作为和慢作为问题。继续减少行政审批事项，简化审批程序，清理行政事业性收费，为项目落地创造公开公平的竞争环境。充分发挥公共行政服务中心和经济合作服务中心作用，对重点项目实行一条龙服务，为项目建设创造良好的服务环境。

（二）坚持“地企融合”、“双轮驱动”，进一步扩大对外开放

积极推进全面开放，大力引进更多的中省直、本地民营、外埠民营及境外企业资本集聚鞍山，实现融合发展。

大力支持鞍钢发展。鞍钢是鞍山经济发展的重要支撑，没有鞍钢的发展就没有鞍山的发展。我们要十分珍惜、爱护鞍钢的发展。大力支持鞍钢向世界500强目标迈进。继续为鞍钢发展创造条件，支持鞍钢有取向硅钢、煤焦油深加工等重大项目建设，推进60万吨线材改造、鞍矿二期采选、煤气发生炉等重大项目顺利实施。按照“发展、就业、改革、保障”八字方针，继续推进鞍钢附企等中省直企业厂办大集体改革发展。积极推进鞍钢附企工业园建设，支持鞍钢附企优势企业做大做强。为企业职工提供免费就业培训和用工对接，让更多附企下岗职工就业和转企就业。不断解决附企历史遗留问题，保证改革顺利实施。对大病特困职工继续给予大力关怀和救助。

争取中直大企业投资鞍山。继续支持在鞍中直企业发展，支持中冶东建搬迁改造，打造“百亿东建”；支持中钢热能、中冶焦耐、中冶北方等中直企业发展壮大。继续支持已来鞍投资的中直企业做大做强，推进中船重工、中航科技等企业项目建设。继续加大工作力度，积极争取新的更多的中直企业来鞍投资。

大力吸引域内外、国内外资本涌入鞍山。积极支持和鼓励本地民营企业投资发展，实施民营企业成长工程。积极引进更多的外埠民营企业投资鞍山。大力吸引和发展外资企业。全面拓展招商领域，积极吸引日、韩、欧美、台湾、香港等国家和地区的资本来鞍投资。支持现有外资企业增资扩股，做大做强。

大力发展资本市场。积极引进国内外银行，推进银企合作。积极帮助企业上市。支持股份制科技风险投资公司和租赁、典当公司发展。充分发挥商会、协会作用。积极引导和支持民间资本设立商业性或互助性信用担保机构、风险投资机构。

（三）着力促进城乡一体化发展，加速推进新农村建设

继续坚持一手抓城市、一手抓农村，全面提高新农村建设的重视程度、投资力度和发展速度，切实促进农民增收。市财政投向“三农”资金超过9亿元，增长50%以上。县域地区生产总值和财政一般预算收入增长20%以上。

大力发展以设施农业为重点的现代农业。继续扩大鞍羊路设施农业示范带，建设海城鞍营线和岫岩张庄线设施农业示范带及辽浑太三河滩地牛羊养殖带。全年力争新建温室大棚10万亩、标准化养殖小区150个，新增10个设施农业镇、40个设施农业村，设施农业发展到60万亩，标准化养殖小区发展到600个。政府出资新建34个乡镇动物卫生监督所，实现一乡（镇）一所。实施东部山区引水上山工程，加快发展南果梨等经济林和生态林。以农产品批发市场和产业化龙头企业为牵动，以农村经济合作组织和农村经纪人为纽带，引导广大农民积极参与设施农业建设。全年农业产业化龙头企业发展到200家，农村经纪人达到10000人，新发展农民专业合作组织50个，带动农户增收面超过60%。市财政出资3000万元，组建农业担保公司，帮助农民解决融资难问题。继续实施政府扶持政策，引导金融资本和社会资本加大对农业投入。

大力发展以工业为重点的县域经济。市政府出资4000万元，支持三县一区建设县域工业规划区。继续支持海城电网建设，支持台安发展农产品深加工产业，支持岫岩开展矿产资源勘探，支持千山区城镇化改造。为海、台、岫各建一处变电所。全年县域工业力争新上项目超过500个，新增规模以上企业200家。

大力发展农村现代服务业。加快推进海城中小、台安黄沙、岫岩岫玉、千山宁远等4个大型批发市场的建设和改造。加强西柳服装、南台箱包、宁远蔬菜和千山花鸟鱼等大型批发市场的信息化建设。深度推进“万村千乡”市场工程和“信福工程”，大力发展农村生产资料、生活资料的连锁经营和便民服务，新建2处大型农村消费品配送中心。

全面实施农村人才工程。强化农村人才建设，进一步解决农村发展人才问题。政府出资1000万元，引进、培养农村教育、医疗卫生、动植物防疫和农业技术推广等方面人才，争取每个乡镇卫生院至少有一名全日制本专科毕业的医护人员。大力发展农村职业技术教育，实现农民远程科技培训视频网络村村通。

（四）牢固树立“好字优先”思想，切实转变经济发展方式

坚持经济社会相协调，速度质量效益相统一，改革发展稳定相结合，人与自然相和谐的经济发展方式，在“好”的基础上追求更快发展。切实改变土地先违规后规范、项目先实施后审批、环保先污染后治理、安全先生产后整改的发展方式，走出一条科技含量高、能耗污染低、安全保障好、经济发展“好中求快”的新路子。

加速提升自主创新能力。落实科技兴市战略，提高自主创新能力，推进创新型城市建设。投资建设鞍山自主创新成果产业化基地。强化企业主体地位，实施培育科技创新型企业计划，培育和发展国家和省级技术创新示范企业，企业与高校联建研发中心、中试和产业化基地，重点实施重大科技攻关项目。

全面扎实推进节能减排。推进结构节能减排。严格执行能源消耗评价制度和环境评价制度，坚决不上高耗能、高污染项目。推进技术节能减排。市政府设立1500万元节能减排专项资金，支持企业节能改造、关键技术攻关和节能技术应用。年内所有燃煤电厂全部安装脱硫设施。推进管理节能减排。全面开展污染源普查工作，加大对重点污染源的监测和污染治理设施的监管。按照整合重组、搬迁改造、关停并转三条途径，解决小炼钢、小采矿、小选矿、小化工、小印染、小水泥等企业问题。加快取缔建成区10吨以下燃煤锅炉。城市生活污水处理率力争超过70%。

切实加强安全生产和土地管理。加大安全生产、消防安全的监管和执法力度。进一步加大隐患排查和整改力度，不达标的企业一律限期整改。严格责任追究制度。落实和强化企业在安全生产工作中的主体地位和责任。坚持依法征地、依法供地、依法用地，不断完善土地管理长效机制。

（五）统筹城乡建设与管理，努力改善城乡环境

按照创建全国文明城市的总体要求，着力提升城市功能。严格执行《城乡规划法》，做到城乡统一规划，统筹建设。

加强城乡基础设施建设。在城市，继续改造完善城市路网。年内建成二环线，将灵山路东延至齐大山形成北外环；北起灵山路，南至陈家台，新建东外环；将旧腾路东延至矿工路形成南外环；将腾达大道南延至旧腾路形成西外环。将园林路南延至南外环，改造中华路、人民路。加快建设和改造城市桥涵。新建深营路东线隧道和曙光路沙河大桥，改造虹桥、民生路地道桥、陈家台大桥、站前地下商场和停车场。完成第二污水处理厂二期工程建设，开工建设高新区南沙河污水处理厂。开通鞍山至北京民用航线。桃仙机场鞍山候机楼投入使用。继续推进街巷路和小区改造。在农村，市政府出资3000万元，支持海城、台安、岫岩县城改造。出资2000万元，支持海城和岫岩实施腾村建区，拓展矿产品深加工区，集中建设小城镇，改善群众居住环境。在实现村村通油路基础上，逐步实现村内通油路。支持哈大高速客运专线、丹海高速公路和岫庄铁路建设。

加强城乡生态环境建设。在城市，北部整治南沙河，南部治理杨柳河，形成南北两条城市内河，打造碧波荡漾的新景观。南沙河全面清淤，新建三条橡胶坝，沿河建设滨河路、带状公园和劳动公园；综合整治杨柳河周边环境，全面关停沿岸小选矿、小采矿等排污企业，疏通河道，新建一座橡胶坝，沿线实施水系改造和绿化、亮化。继续推进二一九、孟泰、永乐、烈士山、静湖和湖南等公园改造，加强玉佛山、千山、汤岗子等景区建设和管理。在千山旅游度假区、高新区东区等南沙河沿线实施雨污分离，污水集中处理，净流排入南沙河。大力推进矿山复垦，对东鞍山、西鞍山、大孤山面积达10平方公里的矿山排岩场稳定部分，实施复垦和抑尘工程。推进城区主次干道两侧绿化，新增城市绿地100万平方米。开展千山水库规划论证。加速减少城区自备井。在农村，支持海城、岫岩开展矿山复垦。继续实施千村绿化工程。保护好大麦科、三岔河等湿地，加强辽、浑、太三河流域治理及台安西北部地区防风固沙建设。

加强城乡管理。在城市，继续开展以“整治经营秩序、规范城市管理”为主要内容的专项行动。规范收购站、煤炭、建材等行业经营行为。下力量治理无照经营、乱停乱放、乱搭乱建等违法违章行为，逐步取缔马路市场及占道经营，推进退路进厅。规范城市机动车和非机动车管理，打击非法营运行为。加强对货运车辆管理，改善市区交通状况。清除城市小广告，粉饰美化临街建筑，搞好山体、桥涵和主要建筑物亮化，提升城市品位。在农村，将城市管理向农村延伸，加强镇街环境卫生管理，继续开展村屯“四清四改”。

（六）切实解决城乡民生问题，全面提高人民生活水平

由政府出资，在就业、保障、住房、供水、燃气等方面继续为城乡群众办实事，让城乡人民学有所教、劳有所得、病有所医、老有所养、住有所居，共同走上幸福生活之路。

继续解决好城乡居民住房问题。在城市，加快回迁房建设，确保城区棚户区和危房改造动迁的居民按时回迁。新建1000套低价住房和500套廉租周转房，解决低收入家庭住房。加快大孤山、齐大山和眼前山三大矿区危旧房改造，改善矿区居民的居住条件。建设劳模大厦和军人之家，努力解决劳动模范和驻鞍现役军人住房问题。加大住房货币化分配投入，加速解决机关事业单位在职和离退休职工及企业离休干部的住房问题。在农村，推进地处城乡结合部的平房区改造。加快推进工业规划区内村屯的整体移迁。加大县域棚户区和危房改造力度。通过腾村建区，加快改善海城、岫岩矿区群众的居住条件和生活环境。帮助农村3000户困难家庭解决住房问题。

认真解决城乡就业问题。在城市，大力开发就业岗位，促进就业再就业可持续发展，全年实名制就业超过10万人，保证“零就业家庭”动态为零，城镇登记失业率控制在4.5%以内。认真落实鼓励大学生就业政策，解决大中专毕业生就业难题。做好部队转业干部、退伍士兵和军嫂的就业安置。建成市劳动力市场，在每个县（市）区各建一处劳动力市场。在农村，搞好农民免费技能培训，让更多被征地农民在工业规划区内企业就业，引导农村剩余劳动力进城务工，有组织地开展劳务输出。

继续完善城乡社会保障体系。在城市，以贯彻落实《劳动合同法》为契机，全面推进有单位人员社会保险应保尽保和应缴尽缴。将城市合作医疗转变为城镇居民基本医疗，提高医疗保障水平，建立全覆盖的医疗保障体系。提高城市最低工资标准，从每月500元提高到580元；继续提高企事业单位离退休人员养老金标准；提高低保家庭老年人和丧失劳动能力者保障标准，保证困难居民基本生活。对城市低保对象实行两年一次免费体检。在农村，加大政府投入，在完善被征地农民养老保险制度基础上，逐步建立健全惠及广大农民的养老保险和社会福利制度。政府出资，将农村新型合作医疗筹资标准由50元提高到100元。

继续改善城乡居民供暖、供气、供水和出行条件。在城市，继续加大铁西等地区供暖管网和分户改造力度。利用煤制气项目，解决城市气源问题。利用大伙房供水和引汤入鞍二期供水，改善城区居民饮用水质量。大力发展城市公共交通，新购110辆城市公交车辆，进一步方便群众出行。在农村，全面解决剩余30多万农民饮水安全问题，使鞍山成为全国第一个农村全部用上自来水的城市。出资6000万元，为50个村建设生物质燃气站。鼓励社会参与，发展农村公共交通。

强化消费品价格和食品药品安全管理。全力扶持粮食、肉类、蔬菜、食用油等主要农副产品生产，增加粮食和肉类等商品储备，保证市场商品供应。加强市场价格监管，严厉查处违价行为。市政府不出台新的涨价政策，保持物价基本稳定，适当提高大学生食堂、困难群体生活补助。全市居民消费价格指数力争低于全省平均水平。不断加强食品药品生产、运输、仓储和销售的全过程监管，严厉打击不法行为，防止假冒伪劣产品流入市场，切实保证食品药品安全。

（七）以争创全国文明城市为统领，加快推进城乡社会建设

大力推进社会事业全面进步，加快城乡社会事业一体化发展，建设文明和谐鞍山。

开展创城攻坚战。全面落实创城任务，重点解决文明礼仪、公共秩序、社会服务、市容市貌、市场环境等领域存在的突出问题。深入开展“迎奥运、讲文明、树新风”等精神文明创建活动，加强思想道德教育，提高市民文明素质，形成知荣辱、讲文明、促和谐的良好风尚。办好千山国际旅游节。加强师德医德等职业道德、社会公德和家庭美德教育。宣传和弘扬鞍山人精神，推进诚信鞍山建设，全面提高城市文明程度。

推进城乡教育均衡发展。加大政府投入，支持辽宁科技大学和鞍山师范学院做大做强，打造成省内乃至东北名校。在城市，免收中小学义务教育阶段学杂费，全面提升义务教育水平。整合职业教育资源，建设鞍山职业教育基地。增加财政投入，减轻高中偿贷压力。在农村，全面改造中小学危房，在有条件的乡镇加速建设中小学寄宿制学校，对不具备条件的乡镇，配备校车或提供交通补贴，解决农村学生上学难问题。在七个县（市）区各设一所免费高中，提高高中普及率。

发展城乡文化事业。在城市，加大政府投入，支持文化团体发展。加强文化设施建设，市博物馆、群众艺术馆投入使用，规划建设市科技图书馆。改造市图书馆、少儿图书馆和盲文图书室。扩建市档案馆、方志馆。在农村，支持各县（市）区建设文化馆、图书馆。年内每个村都有1个文化室，三年内每个乡镇各建1所不小于300平方米的文化站。

发展城乡医疗卫生事业。在城市，坚持发展公共医疗卫生事业，整合市区医疗卫生资源。加快社区卫生服务中心建设。在农村，加大农村医疗卫生事业投入，大力发展县乡公立医院，尤其是中心乡镇公立医院。加快改造乡镇卫生院设施，配备乡镇医院和村卫生所装备，让农民“大病不出县，一般病不出镇，小病不出村”，为城乡人民群众提供安全、有效、方便、价廉的医疗卫生服务。

发展城乡体育事业。做好迎奥运的各项准备工作，组织好奥运火炬传递。规划建设鞍山奥林匹克中心，创立鞍山市乒乓球学校，进一步提高竞技体育水平。将各城区中小学校操场清晨向市民开放，年内每个村都有1处健身广场，配齐健身器材，为城乡群众提供更多的健身场所。

切实加强社会管理。深入开展和谐社区创建活动。继续加强公安机关“三基”工程建设，推进消防、武警训练中心建设，提升平安钢都建设水平。完善市区两级法律服务体系。高度重视信访工作，推进市长信箱进社区，畅通人民群众利益诉求渠道，建立并形成以解决实际问题为主的信访工作新机制，切实维护好人民群众的合法权益。

加快发展老龄事业，改造市老干部活动中心和建设市县（区）老年人活动中心，改造20所农村敬老院。实施免费婚检，大力完善市县（区）计划生育服务中心。高度重视残疾人事业，认真解决残疾人就业问题，提高残疾人生活水平。继续做好双拥工作，推进驻军后勤保障社会化。进一步做好新闻出版、广播电视、民族、宗教、外事、侨务、对台、接待、气象、地震、人防、民兵预备役等方面工作。

（八）全面加强政府自身建设，努力打造法治政府

进一步落实《依法行政实施纲要》，继续推进政府管理创新和制度创新，努力建设廉洁、勤政、务实、高效政府。

加强民主法制建设。自觉接受人大依法监督，坚持向人大及其常委会报告工作制度，认真落实人大及其常委会的各项决议。自觉接受政协民主监督，主动与人民政协民主协商，将政治协商纳入政府决策程序。认真办理人大代表建议和政协提案。围绕民生、公共服务和社会管理等方面加强行政立法，开展行政立法实施效果考评。深入推进“五五”普法。积极支持工会、共青团、妇联、科协等群团组织开展工作。

加强政府制度建设。健全政府科学民主决策机制，切实提高政府决策科学化、民主化水平。继续加强国有资产监督管理，建立国有资本经营预算制度，对重点企业实行绩效考核。健全完善政府采购、政府投资建设项目招投标、产权交易等制度建设。继续推进综合行政执法改革。全面推进政务公开。

加强机关作风建设。健全政府机关绩效考核机制，提高政府工作效能。严格执行领导干部廉洁从政各项规定，坚决遏制腐败现象和不正之风。加强队伍建设，提高公务员队伍的整体素质。继续保持只争朝夕、埋头苦干、廉洁奉公、艰苦奋斗的工作作风。

各位代表，回顾过去，加速经济隆起、构建和谐鞍山，我们已经迈出坚实步伐；展望未来，全面振兴鞍山老工业基地，我们责任在肩、任重道远。让我们在党的十七大精神指引下，在市委的领导下，在市人大、市政协监督支持下，万众一心，开拓进取，为实现鞍山老工业基地全面振兴，为钢城人民更加幸福而不懈奋斗！
